# Time-dependent mRNA expression of selected pro-inflammatory factors in the endometrium of primiparous cows postpartum

**DOI:** 10.1186/1477-7827-8-152

**Published:** 2010-12-22

**Authors:** Christoph Gabler, Claudia Fischer, Marc Drillich, Ralf Einspanier, Wolfgang Heuwieser

**Affiliations:** 1 Institute of Veterinary Biochemistry, Freie Universität Berlin, Oertzenweg 19b, 14163 Berlin, Germany; 2 Clinic for Animal Reproduction, Freie Universität Berlin, Königsweg 63, 14163 Berlin, Germany; 3 Clinic for Ruminants, Section for Herd Health Management, Vetmeduni Vienna, Veterinärplatz 1, 1020 Wien, Austria

## Abstract

**Background:**

Inflammatory processes and infections of the uterine wall must be accepted as a physiological event in dairy cows after calving. This might result in clinical or subclinical endometritis which is assumed to impair reproductive performance in the current lactation. Several cytokines and acute phase proteins have been discussed as local and systemic mediators of these inflammatory processes. The aim of the present study was to investigate the endometrial mRNA expression of the chemokine CXC ligand 5 (*CXCL5*), interleukin 1β (*IL1B*), *IL6*, *IL8*, tumour necrosis factor alpha (*TNF*), prostaglandin-endoperoxide synthase 2 (*PTGS2*) and haptoglobin (*HP*) in the postpartum period.

**Methods:**

Endometrial samples were obtained from primiparous cows (n = 5) on days 10, 17, 24, 31, 38 and 45 postpartum (pp) using the cytobrush technique. Cytological smears were prepared from cytobrush samples to determine the proportion of polymorphonuclear neutrophils (PMN). Total RNA was extracted from endometrial samples, and real-time RT-PCR was performed.

**Results:**

A time-dependent mRNA expression of the investigated factors was found for the course of the postpartum period. In detail, a significantly higher expression of these factors was observed on day 17 pp compared to day 31 pp. Furthermore, the proportion of PMN peaked between days 10-24 pp and decreased thereafter to low percentages (< 5%) on day 31 pp and thereafter. In addition, *CXCL5*, *IL1B*, *IL8 *and *HP *mRNA expression correlated significantly with the proportion of PMN (P < 0.05). A significantly higher *CXCL5*, *IL1B*, *IL6*, *IL8*, *PTGS2 *and *TNF *mRNA content was observed in samples from cows with an inflamed endometrium compared with samples from cows with a healthy endometrium (P < 0.05).

**Conclusions:**

These results show that inflammatory cytokines and acute phase proteins are expressed in the bovine endometrium in a time-related manner during the postpartum period, with a significant expression peak on day 17 pp as a possible mucosal immune response in the uterus. The evaluation of the expression patterns of such candidate genes may reveal more information than only determining the percentage of PMN to judge the severity of an inflammation.

## Background

Invasion of aerobic and anaerobic bacteria into the uterus is common in dairy cattle after parturition. The uterine bacterial colonisation is non-specific and includes a variety of pathogenic bacteria, e.g. *Escherichia coli, Arcanobacterium pyogenes, Fusobacterium necrophorum *and *Prevotella species*. Within 2-4 weeks after parturition most of the bacteria are eliminated spontaneously [1]. Depending on the pathogenicity of bacteria, bacterial load, responsiveness of the immune system and other risk factors, infection may result in acute metritis or, if persistent, in clinical endometritis. Clinical endometritis is defined as an inflammation of the endometrium identified by the presence of purulent or mucopurulent discharge detectable in the vagina later than 21 days postpartum (pp), and is not accompanied by systemic signs [2].

The first response of the innate immune system is the invasion of neutrophils to the site of bacterial infection, i.e. the endometrium. This increase of polymorphonuclear neutrophils (PMN) is used to define and diagnose subclinical endometritis [3,4]. Subclinical endometritis is described as an inflammation of the uterine wall in clinically healthy cows, i.e. in cows with no vulvar discharge. To detect subclinical endometritis, cytological samples can be obtained from the uterus by low-volume uterine lavage or by using the cytobrush technique [3,4]. Clinical as well as subclinical endometritis impair reproductive performance resulting in decreased conception rates and prolonged days open [3-5], leading to economic losses to the dairy farmers. However, the treatment of subclinical and clinical endometritis is still under discussion. Additional information about the underlying mechanism of inflammation is needed to understand the pathology of these diseases and to develop new therapeutic approaches in the future.

The invasion of PMN determined by cytology describes only the cellular reaction to an inflammatory impact. Therefore, pro-inflammatory enzymes and cytokines have been suggested to be synthesized as mediators for inflammatory processes. A variety of factors are secreted to chemoattract other immune cells and to control the inflammatory response. Furthermore, the epithelium is also involved in the innate immune response against bacteria. Bovine endometrial epithelial cells express Toll-like-receptors that recognise specific bacterial components [6].

It is apparent that a complex and well-balanced network of cytokines is present in the uterus [1,7]. Bacteria that invade through the open cervix after parturition and persist in the uterus can disturb this fine-tuned system. Inflammatory responses may lead to perturbation of hypothalamic and pituitary function in turn which may contribute to impaired fertility of cows affected with clinical and subclinical endometritis [8].

Potential candidates involved in physiological and pathological events in the bovine endometrium are the chemokine CXC ligand 5 (CXCL5), interleukins 1β, 6 and 8 (IL1B, IL6, and IL8), tumour necrosis factor alpha (TNF), prostaglandin-endoperoxide synthase 2 (PTGS2), and haptoglobin (HP) [7]. Real-time RT-PCR has revealed that *CXCL5*, *IL1B*, *IL8 *and *TNF *mRNA are significantly higher expressed in the endometrium of cows with subclinical or clinical endometritis than in healthy cows [7].

Therefore, the aim of the present study was to elucidate processes taking place in the bovine endometrium during the postpartum period on a molecular basis. Focus of the investigation was the mRNA expression of *CXCL5*, *PTGS2*, *HP*, *IL1B*, *IL6*, *IL8 *and *TNF*. Specific objectives were to analyse the selected mRNA expression patterns in endometrial samples from primiparous cows: 1) in a time-course related manner starting on day 10 pp until day 45 pp and 2) in correlation to the proportion of PMN in the cytological samples and to the health status (healthy endometrium versus inflamed endometrium).

## Methods

### Collection of endometrial samples during the postpartum period

Holstein Friesian heifers (n = 5) were housed at the Clinic for Animal Reproduction, Freie Universität Berlin. All heifers calved without assistance. Fetal membranes were expelled within 12 h after calving. Rectal temperature was measured daily. None of the cows showed signs of acute metritis. All cows were examined by palpation of the uterus per rectum and vaginoscopy starting on day 10 pp. The first endometrial samples from each cow were collected on that day. Further examination of cows and sampling was performed on days 17, 24, 31, 38 and 45 pp, respectively.

Endometrial samples for cytological assessment and mRNA analysis were collected from the uterus using the cytobrush technique [9,10]. Briefly, a cytobrush (Cytobrush plus GT, Medscand, Malmö, Sweden), screwed onto a 70-cm long rod, protected by a metallic catheter, was inserted into the uterine body via the cervix. This approach required only one passage through the cervix. Three different samples per cow were taken by introducing a new cytobrush through the protection catheter for each collection. Cells were collected by rotating the cytobrush clockwise while in contact with the uterine wall.

Cell samples from two cytobrushs per animal and day were transferred into two separate reaction tubes containing RNAlater (Sigma, Deisenhofen, Germany). One cytobrush served for cytological analysis. The brush was rolled onto a sterile glass microscope slide, fixed and stained with the Hemacolor staining set (Merck, Darmstadt, Germany) following the manufacturer's protocol. A total of 300 cells were counted under a microscope (x 400 magnification) to determine the proportion of PMN.

These data served to classify the health status of the uterus. Two criteria were applied to define an inflammation of the endometrium: presence of purulent or mucopurulent vaginal discharge detected by palpation of the uterus per rectum and vaginoscopy (criteria in the literature for the definition of clinical endometritis [2]) and/or percentage of more than 5% of PMN in the cytological sample (criteria in the literature for the definition of subclinical endometritis [3,10]). The uteri without signs for inflammation (no mucopurulent/purulent discharge and <5% PMN in the cytological sample) were defined as healthy.

Blood samples were drawn on the days of cell collection. The serum concentrations of progesterone and 17β-oestradiol were determined using a chemiluminescence detection system (Immulite, Diagnostic Products, Los Angeles, CA, USA) on an automated analyser (Immulite 2000, DPC Biermann, Bad Nauheim, Germany) at the Institute for Zoo and Wildlife Research (Berlin, Germany).

In addition, ovaries and uterus were examined by transrectal ultrasonography with a 5 MHz linear array transducer (Pie Medical, Maastricht, Netherlands), and results were documented.

### Total RNA extraction and reverse transcription

Harvested endometrial cell samples were subjected to RNA extraction using Invisorb Spin Cell RNA Mini Kit (Invitek, Berlin, Germany) as previously described in detail [10]. Isolated total RNA was quantified photometrically at a wavelength of 260 nm. Quality of RNA was verified by loading 1 μL of total RNA onto a RNA 6000 Nano Chip using the Agilent 2100 Bioanalyzer (Agilent, Waldbronn, Germany) following the manufacturer's instructions.

Single stranded cDNA was generated out of 0.5 μg total RNA using 200 U Moloney Murine Leukemia Virus reverse transcriptase (M-MLV RT) (Fermentas, St. Leon-Rot, Germany) and 3.75 μM random hexamers (Amersham Bioscienes, Freiburg, Germany) in a total volume of 40 μL [11]. The cDNA obtained was stored at -20°C until further analysis. Before reverse transcription, a genomic DNA removal by DNase digestion was performed. To monitor the absence of genomic DNA or other contaminations, reactions containing RNA samples without M-MLV RT or template (H_2_O) were carried out at the same time as negative controls.

### Real-time PCR

For mRNA quantification of the selected factors, real-time PCR was performed as previously reported using the primer pairs listed in Table 1[11].

**Table 1:** Primer sequences

Gene	Primer sequence	Reference	Fragment size (bp)	Annealing temperature
***CXCL5***	for 5'-TGA GAC TGC TAT CCA GCC G-3'	[7]	193 bp	61°C
	rev 5'-AGA TCA CTG ACC GTT TTG GG-3'			
***IL1B***	for 5'-CAA GGA GAG GAA AGA GAC A-3'	[29]	236 bp	56°C
	rev 5'-TGA GAA GTG CTG ATG TAC CA-3'			
***IL6***	for 5'-TCC AGA ACG AGT ATG AGG-3'	[29]	236 bp	56°C
	rev 5'-CAT CCG AAT AGC TCT CAG-3'			
***IL8***	for 5'-CGA TGC CAA TGC ATA AAA AC-3'	[7]	153 bp	56°C
	rev 5'-CTT TTC CTT GGG GTT TAG GC-3'			
***PTGS2***	for 5'-CTC TTC CTC CTG TGC CTG AT-3'	[11]	359 bp	60°C
	rev 5'-CTG AGT ATC TTT GAC TGT GGG AG-3'			
***HP***	for 5'-TGG TCT CCC AGC ATA ACC TC-3'	[7]	217 bp	60°C
	rev 5'-TTG ATG AGC CCA ATG TCT ACC-3'			
***TNF***	for 5'-CAA GTA ACA AGC CGG TAG CC-3'	[30]	354 bp	60°C
	rev 5'-GCT GGA AGA CTC CTC CCT G-3'			
***18S rRNA***	for 5'-GAG AAA CGG CTA CCA CAT CCA A-3'	[11]	337 bp	61°C
	rev 5'-GAC ACT CAG CTA AGA GCA TCG A-3'			

Gene transcripts, primer sequences and annealing temperatures used for real-time PCR with resulting amplicon length.

In short, 1 μL cDNA was subjected to real-time PCR in a total volume of 10 μL containing 0.4 μM of each primer (forward and reverse) and 1x SensiMix (dt) (Quantace, Berlin, Germany). Amplification was done using the Rotor-Gene 3000 (Corbett Research, Mortlake, Australia) and the following parameters: a denaturation step at 95°C for 10 minutes, a three-step cycling amplification including denaturation at 95°C for 15 sec, annealing at the indicated temperature in Table 1 for 20 sec and extension at 72°C for 30 sec, a melting curve program (50-99°C) with continuous fluorescence measurement and a final cooling step to 40°C. Data acquisition was carried out at the end of each extension step at 72°C. A total of 45 cycles were performed (exception 18S rRNA: 25 cycles). A dilution series of the according PCR products with known concentrations was amplified simultaneously with the samples as a standard. Quantities of specific mRNA were calculated in comparison with the standard curves using Rotor-Gene 6.0 software. The obtained melting points of the amplified PCR products confirmed specific amplification. Negative controls containing no template (H_2_O) or non reverse-transcribed RNA did not show a signal verifying that obtained amplicons were not derived from contaminations or genomic DNA.

### Statistical analysis

Gene expression of the investigated factors obtained by real-time PCR was normalised. To this end, absolute quantities of each gene were divided through the quantities of 18S rRNA in each sample. Means were obtained from the independently processed duplicates for each animal and collection time. Normalised values were analysed by the Kruskal-Wallis-H-test. When this test indicated significant differences, the Mann-Whitney-U-test was used to compare the samples from day 17 pp with day 31 pp. These points in time were chosen because of the results obtained from cytological analysis (day 17 pp: high PMN content; day 31 pp: first point in time of low PMN content). Furthermore, expression patterns of the selected factors were analysed by comparing samples from an inflamed endometrium with samples obtained from cows with a healthy endometrium using the Mann-Whitney-U-test.

The Pearson's test was used to calculate the correlation between each of the factors as well as between the percentage of PMN and pro-inflammatory factors.

All statistical calculations were performed by using SPSS for windows version 18.0 (SPSS, Chicago, IL, USA). Values of P < 0.05 were considered to be significant.

## Results

### PMN content in cytobrush samples and clinical observations

**Figure 1:**
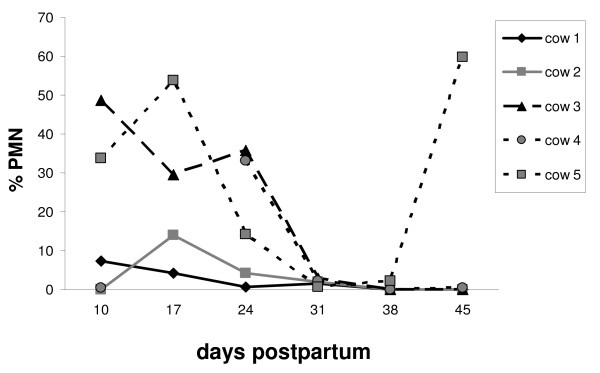
**PMN content in endometrial samples**. PMN content in bovine endometrial samples collected during the postpartum period on days 10, 17, 24, 31, 38 and 45, respectively (n = 5 animals). Sample from cow 4 on day 17 pp is missing due to technical reasons.

The assessment of the cytological samples revealed that the PMN content changed in a time-dependent manner. In detail, the PMN content, which was > 5% during the postpartum period (on day 10 pp or day 17 pp), declined to percentages < 5% on day 31 pp (Figure 1). During the first 2-3 weeks of the puerperium, individual differences were wide, especially on day 10 pp. Some animals had a content of less than 5% PMN in their cytological sample, while others revealed values higher than 30%. In general, however, a PMN content higher than 5% was associated with vaginal discharge (except for two samples) (Table 2). In the later postpartum phase (day 31-45 pp), most animals showed neither vaginal discharge nor an increased proportion of PMN. One cow (animal 5) showed mucopurulent vaginal discharge from day 10 pp until day 38 pp (diagnosed from day 31 pp on as clinical endometritis) which resulted in an elevated PMN level on day 45 pp and a persisting subclinical endometritis. Out of 29 samples obtained from the cows over the sampling period, 16 samples were classified as "healthy", and 13 samples were considered obtained from an "inflamed endometrium".

**Table 2:** Clinical observations

Animal	Day pp	Clinical Observation
**1**	10	vaginal discharge; reddish-brown
	17	w/o. cl. ob.
	24	w/o. cl. ob.
	31	w/o. cl. ob.
	38	w/o. cl. ob.
	45	w/o. cl. ob.
**2**	10	w/o. cl. ob.
	17	w/o. cl. ob.
	24	w/o. cl. ob.
	31	w/o. cl. ob.
	38	w/o. cl. ob.
	45	w/o. cl. ob.
**3**	10	vaginal purulent discharge; reddish-brown
	17	vaginal purulent discharge
	24	vaginal purulent discharge
	31	w/o. cl. ob.
	38	w/o. cl. ob.
	45	w/o. cl. ob.
**4**	10	w/o. cl. ob.
	17	no PMN counted; w/o. cl. ob.
	24	vaginal discharge
	31	w/o. cl. ob.
	38	w/o. cl. ob.
	45	w/o. cl. ob.
**5**	10	vaginal discharge; reddish-brown
	17	vaginal purulent discharge
	24	vaginal mucopurulent discharge
	31	vaginal mucopurulent discharge
	38	vaginal mucopurulent discharge
	45	w/o. cl. ob.

Clinical observations of cows during the postpartum period (pp: postpartum; w/o. cl. ob.: without clinical observation).

The observations from ultrasonography, adspection and palpation as well as the serum concentrations of progesterone (P_4_) and 17β-oestradiol (E_2_) were used to allocate the cows in the postpartum period to distinct oestrous cycle phases. Only cow 2 was in the luteal phase on day 45 pp (corpus luteum detected and >1.5 ng P_4_/ml serum), and none of the cows were in oestrus at sample collection (signs of oestrus and >2 pg E_2_/ml serum).

Extracted RNA from all samples harvested by the cytobrush method was of a good quality, evaluated by standard methods (data not shown) as previously demonstrated [10]. The two independently processed samples of each cow revealed a similar mRNA expression pattern for each investigated factor (median of variation 30%).

### Expression of *CXCL5 *mRNA

**Figure 2:**
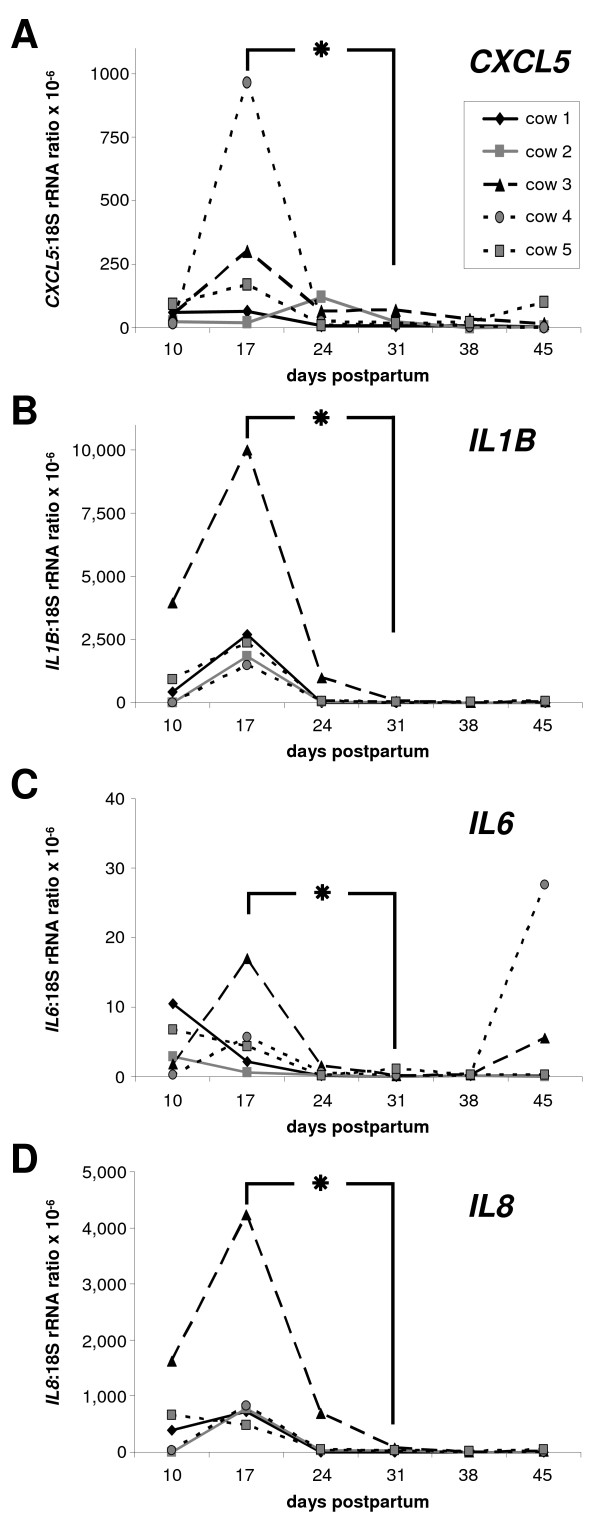
**mRNA expression of *CXCL5*, *IL1B*, *IL6 *and *IL8 *in endometrial samples**. mRNA expression of *CXCL5 *(**A**), *IL1B *(**B**), *IL6 *(**C**) and *IL8 *(**D**) in bovine endometrial samples collected during the postpartum period on days 10, 17, 24, 31, 38 and 45 pp, respectively (n = 5 animals). The contents of each mRNA are expressed as ratio relative to individual 18S rRNA values as an internal control. The asterisk indicates significant differences between day 17 pp and day 31 pp; P < 0.05.

The expression pattern of *CXCL5 *mRNA in bovine endometrial samples varied during the postpartum period (Figure 2A). In general, *CXCL5 *mRNA expression was higher in samples obtained from an inflamed endometrium compared with the healthy endometrium (P < 0.05; mean of normalised expression ± S.E.M. × 10^-6^: 74 ± 23 and 25 ± 8, respectively). The mean expression of *CXCL5 *mRNA was significantly higher on day 17 pp than on day 31 pp. The endometrial sample of one animal (cow 5) showed an increase of *CXCL5 *mRNA expression on day 45 pp, which was accompanied by an elevated PMN level (Figure 1). The range of *CXCL5 *mRNA expression in endometrial cell samples during the postpartum period was 64-280 fg/μg total RNA.

### Expression of *IL1B*, *IL6 *and *IL8 *mRNA

The expression patterns in the luminal endometrium of *IL1B *and *IL8 *were almost identical during the postpartum period, in contrast to *IL6 *(Figure 2B-D). However, the mRNA expression of these selected interleukins was significantly higher (P < 0.05) in samples from an inflamed endometrium than in samples from a healthy endometrium (mean of normalised expression ± S.E.M. × 10^-6^; *IL1B*: 1594 ± 776 and 178 ± 168; *IL6*: 3.7 ± 1.4 and 2.3 ± 1.7; *IL8*: 693 ± 322 and 57 ± 45, respectively).

In detail, an increase of *IL1B *mRNA expression was observed from day 10 pp to the highest levels on day 17 pp (Figure 2B). Thereafter, a significant decrease of *IL1B *mRNA content was noted up to day 31 pp. After this point, *IL1B *mRNA expression was very low or not detectable.

In contrast, mRNA expression of *IL6 *was inconsistent (Figure 2C). Average *IL6 *mRNA contents were significantly higher on day 17 pp compared with day 31 pp (P < 0.05). The *IL6 *mRNA content declined after day 17 pp. However, in endometrial samples of two cows an increased *IL6 *mRNA expression was detected on day 45 pp. The PMN content in these cows was <5%.

Pro-inflammatory *IL8 *mRNA was detected in the early phase of the postpartum period with a distinct expression pattern (Figure 2D). As observed for *IL1B*, expression of *IL8 *mRNA was significantly higher in samples obtained on day 17 pp compared with samples collected on day 31 pp. The expression level of *IL8 *was 20-100-fold lower from day 31 pp onwards than on day 17 pp.

One cow (animal 3) showed an approximately fourfold higher expression of all investigated interleukins on day 17 pp compared with the other cows.

The absolute range of mRNA expression for the selected interleukins differed: the highest range was noted for *IL8 *(400-2,000 fg/μg total RNA) followed by *IL1B *(80-400 fg/μg total RNA). *IL6 *expression showed the lowest range, with 4-80 fg/μg total RNA.

### Expression of *TNF *mRNA

**Figure 3:**
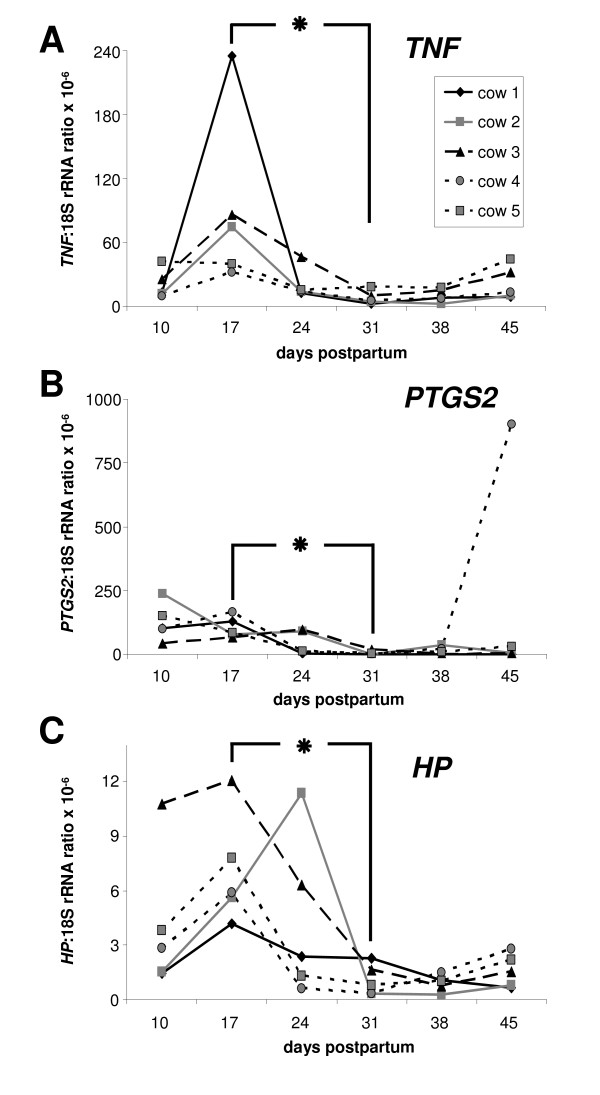
**mRNA expression of *TNF*, *PTGS2 *and *HP *in endometrial samples**. mRNA expression of *TNF *(**A**), *PTGS2 *(**B**) and *HP *(**C**) in bovine endometrial samples collected during the postpartum period on days 10, 17, 24, 31, 38 and 45 pp, respectively (n = 5 animals). The contents of each mRNA were expressed as ratio relative to individual 18S rRNA values as an internal control. The asterik indicates significant differences between day 17 pp and day 31 pp; P < 0.05.

Transcripts for *TNF *were detected in all samples with a time-dependent variation (Figure 3A). The higher expression observed on day 17 pp was significant compared with day 31 pp (P < 0.05). In the late postpartum period, a slight increase of *TNF *mRNA expression was noted in each cow on day 45 pp compared with day 31 pp or 38 pp.

In addition, *TNF *mRNA expression was dependent on the health status. A significantly higher *TNF *mRNA content was observed in samples from cows with an inflamed endometrium compared with samples from healthy cows (P < 0.05; mean of normalised expression ± S.E.M. × 10^-6^: 34 ± 6.7 and 24 ± 14, respectively).

The range of *TNF *mRNA expression in luminal endometrium samples during the postpartum period was 40-160 fg/μg total RNA.

### Expression of *PTGS2 *and *HP *mRNA

Endometrial mRNA expression of *PTGS2 *was found in all samples from day 10-45 pp (Figure 3B). The mean *PTGS2 *mRNA expression declined from day 10 pp to lower levels on day 31 pp and remained on such low expression levels in the late puerperial phase. As observed for the cytokines, this enzyme was also higher expressed on day 17 pp compared with day 31 pp (P < 0.05). One animal (cow 4) showed a dramatic increase of *PTGS2 *mRNA expression on day 45 pp. This was not correlated with an increase of the PMN content but accompanied by an increased *IL6 *mRNA expression. However, the health status correlated with the *PTGS2 *expression, showing higher *PTGS2 *transcript contents in samples from cows with an inflamed endometrium compared with samples from a healthy endometrium (P < 0.05; median of normalised expression × 10^-6^: 6.5 and 0.5, respectively). The range of *PTGS2 *mRNA expression in endometrial cell samples during the postpartum period was 160-800 fg/μg total RNA.

In contrast, the smallest and lowest range of the mRNA expression in the bovine endometrium for all investigated factors in the postpartum period was noted for *HP *(Figure 3C). The absolute amount of *HP *mRNA expression in luminal endometrium samples was in the range of 4-16 fg/μg total RNA. An increase was observed from day 10 pp to day 17 pp. Furthermore, the *HP *mRNA content in the endometrium declined significantly thereafter to about fourfold lower levels on day 31 pp (P < 0.05). There was a tendency towards a slight increase of *HP *mRNA expression on day 45 pp. In contrast to *PTGS2*, no significant difference of the *HP *transcript amount was detected between samples from cows with an inflamed endometrium versus healthy cows.

### Correlation analysis

Significant correlations between the mRNA expression of several factors in endometrial cell samples harvested during the puerperium are listed in Table 3. Especially *IL8 *mRNA contents showed a high correlation with the mRNA expression of all factors except *PTGS2*. The highest correlation was noted between *IL1B *and *IL8 *mRNA expression. *HP *as an acute phase protein showed a high correlation in its local mRNA expression to the mRNA expression of the PMN chemoattractant factors *IL1B *and *IL8*. *PTGS2 *transcript content was only significantly correlated with *IL6 *mRNA expression.

**Table 3:** Correlation coefficients

	*CXCL5*	*IL1B*	*IL6*	*IL8*	*HP*	*PTGS2*	*TNF*	PMN
*CXCL5*	-			0.376	0.397			0.563
*IL1B*		-	0.406	0.983	0.730		0.491	0.424
*IL6*		0.406	-	0.427		0.814		
*IL8*	0.376	0.983	0.427	-	0.718		0.417	0.412
*HP*	0.397	0.730		0.718	-			0.517
*PTGS2*			0.814			-		
*TNF*		0.491		0.417			-	
PMN	0.563	0.424		0.412	0.517			-

Significant correlation coefficients (r) between the mRNA expression of selected pro-inflammatory factors in the endometrial samples collected during the postpartum period on days 10, 17, 24, 31, 38 and 45, respectively (n = 5 animals) presented as a matrix format (P < 0.05). When no value is indicated, no significant correlation between the factors was observed.

In addition, mRNA expression of *CXCL5*, *IL1B*, *IL8 *and *HP *correlated significantly with the proportion of PMN (Table 3). In contrast, a correlation was not observed between the number of PMN and the mRNA expression of *TNF*, *IL6 *and *PTGS2*.

## Discussion

The present study demonstrates a time-dependent presence of PMN in endometrial samples collected until week 8 postpartum. A peak in the proportion of PMN was detected between day 10-24 pp. Furthermore, the percentage of PMN in the cytological samples correlated with an increased mRNA expression of several pro-inflammatory factors. These findings are supported by the literature. The prevalence of subclinical endometritis, defined by an increased proportion of PMN, in a time-dependent manner has been previously reported by other authors. Gilbert *et al*. [3] diagnosed 90-100% of the cows examined two and four weeks after parturition by uterine lavage and subsequent cytological assessment as affected with subclinical endometritis. A prevalence of subclinical endometritis of 41% was still noted after two months [3]. In a study using both the cytobrush and lavage techniques for the diagnosis of subclinical endometritis, the percentage of PMN decreased from day 20-33 pp to day 34-47 pp [9].

Our study, although conducted with only five cows, is the first that describes an initial increase of PMN from day 10 pp to day 24 pp, followed by a decrease to percentages about 0% on day 31 pp. This time course of PMN content is in accordance with findings describing a recovery from endometritis or infection within four weeks postpartum [5,12,13]. In the present study, all cows had an increased number of PMN in the uterus in the postpartum period. Thus, it can be hypothesised that a PMN influx is a physiological response to a bacterial infection of the uterus postpartum [14]. Our findings for the presence of PMN and the mRNA expression analysis support previous reports that the diagnosis of clinical or subclinical endometritis should be performed after day 24 pp [15,16].

There was a significant correlation between the presence of PMN and mRNA expression of *IL1B*, *IL8 *and *CXCL5*. These data support evidence that PMN are chemoattracted by substances secreted by epithelial cells and the latter substances are suggested to act together directing PMN to the site of inflammation [17].

In the present study, mRNA expression analysis revealed a significant expression peak on day 17 pp compared with contents on day 31 pp for all investigated factors. This is in agreement with higher expression of CXCL5, IL1B, IL8 and TNF described for several bacterial infections [18-20], indicating that the immune system is activated to eliminate bacteria. Recently, it was suggested that *CXCL5*, *IL1B*, *IL8 *and *TNF *may be suitable markers for the detection of subclinical endometritis because mRNA levels were elevated compared with samples from healthy cows [7]. Interestingly, significant differences in the expression of the selected factors did not exist between samples derived from cows with subclinical and clinical endometritis [7]. This may be explained by the fact that both diseases are an inflammation of the endometrium, only with a different clinical appearance.

The similar expression pattern for *IL1B*, *IL8 *and *CXCL5 *is in accordance with data from the literature stating that IL1B stimulates the production of CXCL5 and IL8 [17,21]. However, it has to be pointed out that the expression of IL1B must occur earlier to stimulate the expression of IL8 and CXCL5, but a weekly sampling can not provide such data. In addition, IL8 and CXCL5 are responsible for the recruitment of PMN [22,23]. In an *in vivo *bovine model, it has been demonstrated that peak numbers of uterine PMN were attracted 6 h after intrauterine application of IL8 [24]. Furthermore, an infiltration of neutrophils into the bovine uterus around ovulation has been reported [25,26]. This was supported by finding an increased mRNA expression of the PMN chemoattractant factors *IL1B*, *IL8 *and *CXCL5 *in the endometrium around ovulation [7]. Therefore, an up-regulated expression of these factors can lead to a subsequent increased influx of PMN.

The mRNA expression pattern of all investigated factors in the endometrial samples of this study was not affected by the presence of steroid hormones because concentrations of P4 and E2 were not elevated before day 45 pp.

The cytokine *TNF *was also expressed in a time-related pattern during the postpartum period. The highest levels on day 17 pp and day 24 pp and the following decline may be explained by the pro-inflammatory response that has been described in the literature [20]. TNF may act as a potent regulator of PGE_2 _as well as of PGF_2α _secretion [27]. TLR stimulation via bacterial lipopolysaccharids leads to a switch from PGF to the PGE series [8]. This has been suggested as a pathogenic mechanism after uterine bacterial infection which could result in an extended luteal phase and thus contribute to an impaired reproductive performance. In this context, *PTGS2*, one of the key-enzymes of the PG synthesis, was up-regulated in the present study in its expression in parallel to *TNF*.

Interestingly, *HP *as an acute phase protein showed a similar local expression pattern as the pro-inflammatory interleukins. In a former study, *HP *was found unspecific for the detection of subclinical endometritis [7].

A recently published study describes the expression of several pro-inflammatory cytokines including *IL1A*, *IL1B *and *IL6 *in the first week postpartum in endometrial biopsies [28]. A higher expression of *IL1 *correlated with decreased fertility parameters. That study compared healthy cows (n = 4) without uterine disorders during the postpartum period and conceiving at the time of first insemination (day 59-74 pp) with cows (n = 4) showing uterine diseases and failing to conceive within 200 days pp. These findings are complementary to the results of the present study and enhance the understanding of events occurring during the postpartum period. The heifers in our study were not selected specifically, and the number of animals did not allow a comparison of reproductive performance data.

## Conclusions

In conclusion, the results of the present study showed that inflammatory cytokines and acute phase proteins are expressed in the bovine endometrium in a time-related manner during the postpartum period with a peak of expression around day 17 pp. Therefore, the evaluation of the expression patterns of such candidate genes reveals more information than only determining the percentage of PMN to judge the severity of inflammation. This knowledge may give further information for new therapeutic strategies, which may be monitored by a local expression analysis of these selected biomarkers. However, this study is only a descriptive and preliminary one. Further investigations with a greater number of cows are required to follow the time-dependent expression profile of such candidate genes in healthy cows, cows developing a subclinical endometritis and in cows showing a clinical endometritis.

## Competing interests

The authors declare that they have no competing interests.

## Authors' contributions

CG, MD, RE and WH planned and devised the experiments. CF conducted the animal study, collected the samples and performed cytological analysis. CF and CG performed the molecular biology analysis. CG, MD, RE and WH wrote the manuscript. All authors read and approved the manuscript.
